# Rescue method using a covered metal stent with an ultra-slim delivery system for failed endoscopic ultrasound-guided rendezvous

**DOI:** 10.1055/a-2695-4501

**Published:** 2025-09-18

**Authors:** Masahiro Yamamura, Hirotoshi Ishiwatari, Akihiro Ohba, Hiroki Sakamoto, Takuya Doi

**Affiliations:** 1 38471 Division of Pancreatobiliary Medicine, Shizuoka Cancer Center, Shizuoka, Japan

Endoscopic ultrasound-guided rendezvous (EUS-RV) is a rescue technique employed when biliary access during endoscopic retrograde cholangiopancreatography (ERCP) fails 1 2 3 4 . However, EUS-RV is challenging due to the bile leakage risk, which can result in fluid collection between the liver and gastrointestinal tract. We report successful EUS-RV rescue using a self-expandable metal stent (SEMS) with an ultra-slim delivery system ( Video 1 ).

A covered metal stent with an ultra-slim delivery system is a useful rescue option after failed endoscopic ultrasound-guided rendezvous, even with fluid accumulation between the liver and gastrointestinal tract.Video 1

A 69-year-old woman developed obstructive jaundice secondary to pancreatic tail cancer. Computed tomography revealed hilar biliary obstruction and intrahepatic bile duct dilation caused by a metastatic tumor. ERCP was initially attempted; however, biliary cannulation failed, prompting same-session EUS-RV. Duodenal puncture was not feasible due to a non-dilated extrahepatic bile duct; therefore, a transgastric approach was adopted. Because the intrahepatic bile duct in segment 2 was less dilated, the dilated duct in segment 3 was punctured using a 19-gauge needle ( Fig. 1 ). A 0.025-inch guidewire was inserted but failed to pass through the hilar biliary obstruction, even when a hydrophilic guidewire was used owing to the highly angulated left hepatic duct ( Fig. 2 ). A double-guidewire technique using a double-lumen cannula was employed 5 . However, bile leakage occurred during catheter exchange, and fluid collection was observed between the liver and stomach, complicating the procedure ( Fig. 3 ).

**Fig. 1:**
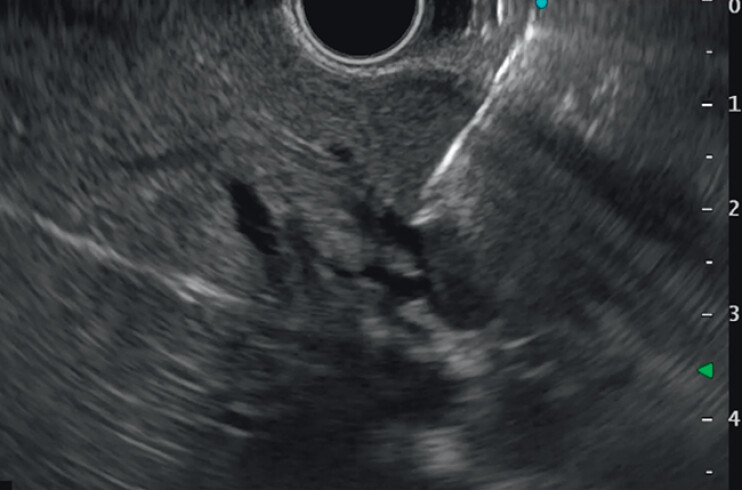
The dilated intrahepatic bile duct in segment 3 was punctured by a 19-gauge needle.

**Fig. 2:**
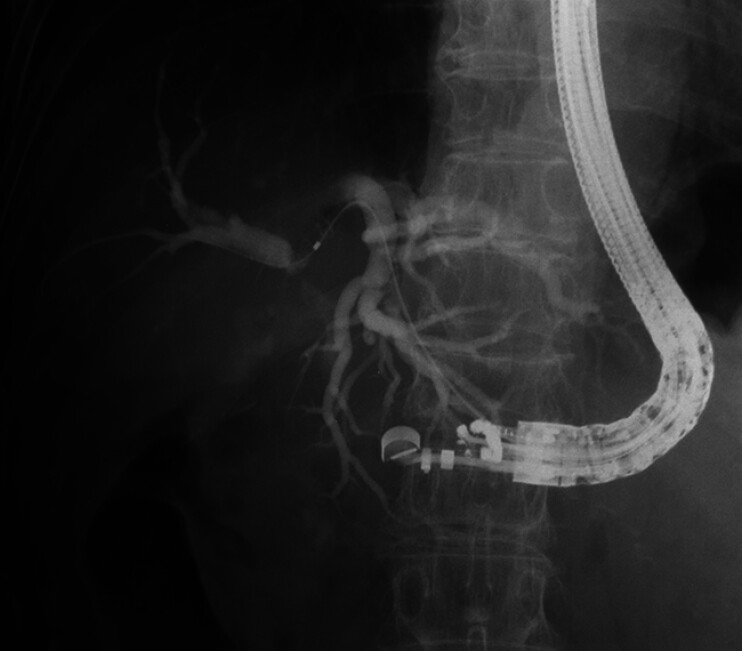
Cholangiography revealed a highly angulated, left hepatic duct. The extrahepatic bile duct was not visualized by contrast, making guidewire insertion even more challenging.

**Fig. 3:**
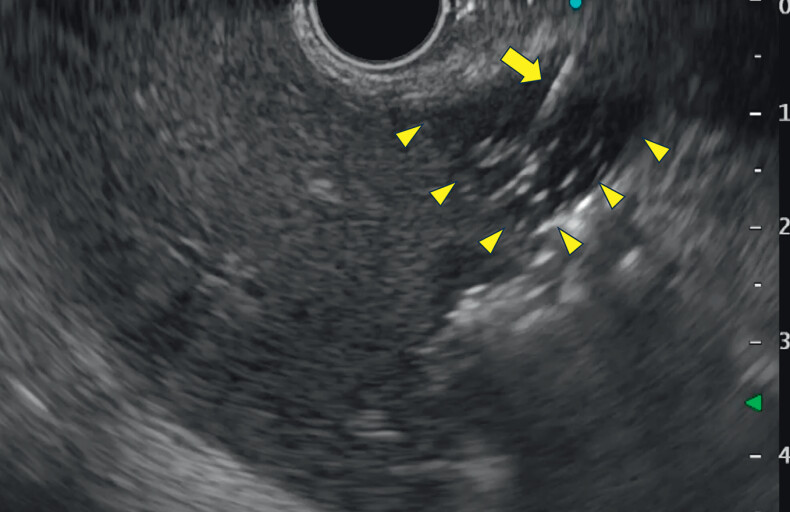
Fluid collection (arrowheads) due to bile leakage was observed between the liver and the stomach. The arrow indicates the guidewire.

We decided to convert the patient to hepaticogastrostomy. Owing to the anticipated technical difficulty in inserting a metal stent with a conventional 8-Fr delivery system without dilation devices such as a cautery dilator or balloon catheter, we selected a fully covered SEMS with a 5.9-Fr delivery system (8-mm × 12-cm HANAROSTENT; M.I. Tech, Seoul, Korea). This facilitated smooth stent insertion, and hepaticogastrostomy was successfully completed ( Fig. 4 ). The patient developed peritonitis, which resolved with conservative treatment. Her total bilirubin levels decreased, and no further biliary drainage was required.

**Fig. 4:**
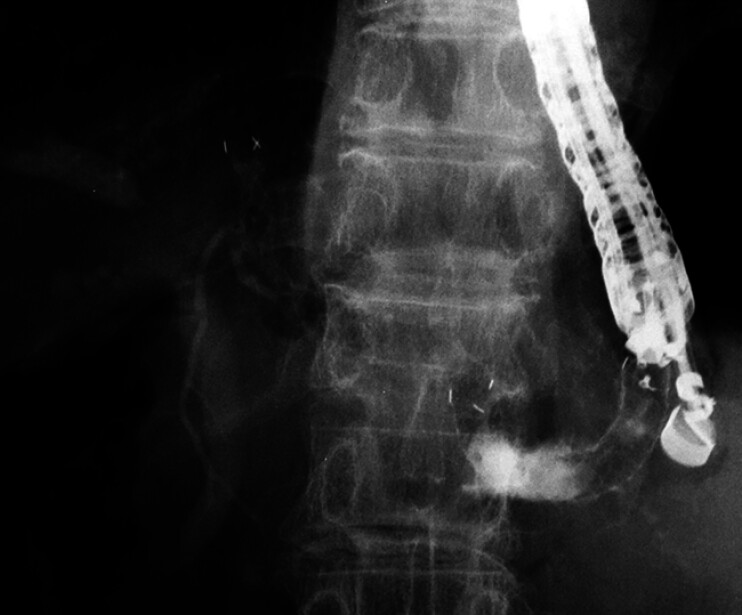
A covered metal stent with a 5.9-Fr delivery system was easily inserted and placed between the liver and the stomach.

Endoscopy_UCTN_Code_TTT_1AS_2AH

## Notes

Endoscopy E-Videos https://eref.thieme.de/e-videos
*E-Videos* is an open access online section of the journal *Endoscopy* , reporting on interesting cases and new techniques in gastroenterological endoscopy. All papers include a high-quality video and are published with a Creative Commons CC-BY license. Endoscopy E-Videos qualify for HINARI discounts and waivers and eligibility is automatically checked during the submission process. We grant 100% waivers to articles whose corresponding authors are based in Group A countries and 50% waivers to those who are based in Group B countries as classified by Research4Life (see: https://www.research4life.org/access/eligibility/ ). This section has its own submission website at https://mc.manuscriptcentral.com/e-videos .
